# Smooth-pursuit performance during Eye-typing from Memory indicates Mental Fatigue

**DOI:** 10.16910/jemr.15.4.2

**Published:** 2022-10-06

**Authors:** Tanya Bafna-Rührer, Per Bækgaard, John Paulin Hansen

**Affiliations:** DTU Management, Technical University of Denmark

**Keywords:** Mental fatigue, smooth-pursuit, eye-tracking, text entry, time-on-task, time of day, attention

## Abstract

Mental fatigue is known to occur as a result of activities related to e.g. transportation, health-care, military operations and numerous other cognitive demanding tasks. Gaze tracking has wide-ranging applications, with the technology becoming more compact and processing power reducing. Though numerous techniques have been applied to measure mental fatigue using gaze tracking, smooth-pursuit movement, a natural eye movement generated when following a moving object with gaze, has not been explored with relation to mental fatigue. In this paper, we report the results from a smooth-pursuit movement based eye-typing experiment with varying task difficulty to generate cognitive load, performed in the morning and afternoon by 36 participants. We have investigated the effects of time-on-task and time of day on mental fatigue using self-reported questionnaires and smooth-pursuit performance, extracted from the gaze data. The self-reported mental fatigue increased due to time-on-task, but the time of day did not have an effect. The results illustrate that smooth-pursuit movement performance declined with time-on-task, with increased error in the gaze position and an inability to match the speed of the moving object. The findings exhibit the feasibility of mental fatigue detection using smoothpursuit movements during an eye-interactive task of eye-typing.

## Introduction

Mental fatigue is defined as a reduction in mental resources and motivation to continue the task, generated by sustained cognitive processing over time (9; 17; 37). Mental fatigue has been emerging as an issue for the able-bodied working population, as well as for people with neuromuscular degeneration such as amyotrophic lateral sclerosis or cerebral palsy and can have severe effects on their daily activities (44; 4; 35). Few studies have assessed mental fatigue during a real-life mental fatigue inducing task, such as during driving (14), hazard detection (30) or a mentally challenging task such as proof-reading (42). We have used a task of eye-typing, a task performed on a daily basis by people with neuromuscular disorders in order to communicate and interact with the world (24), during which we will attempt measuring indications of mental fatigue. We explore the eye-typing interactions using smooth-pursuit eye movements and study the characteristics of the eye movements that depict mental fatigue.

Smooth-pursuit eye movements are natural eye movements (1; 29) generated to visually track a moving object by maintaining a stable image of the object on the retina (12). Smooth-pursuit is a complex process and require cognitive control (12; 6; 27; 5). Moreover, cognitive factors can affect the smooth-pursuit eye movements without voluntary control (41. Thus, eye movements could be utilized to assess these cognitive factors. Mental fatigue, a cognitive factor, is closely associated with cognitive load and is known to affect the ability to focus attention on a task (9; 10), both of which have been shown to influence smooth-pursuit eye movements (21; 26). Current approaches to mental fatigue measurement using eye-based data are based on features such as pupil size (11; 17; 30), saccade velocity and amplitude ( 33; 13), and blink frequency and duration ( 33; 16; 30). However, the effects of mental fatigue on the smooth-pursuit eye movements have not been studied extensively till now (42).

The most commonly used paradigm for mental fatigue measurement involves performing a cognitively challenging task and studying the effect of time-on-task on mental fatigue. Mental fatigue is sometimes accompanied with deterioration in task performance (16; 45; 48), however, in other cases, mental fatigue is a result of an increase in task effort to maintain the task performance (11; 33). Mental fatigue and performance appear to be closely related. Performance on tasks, such as a simple search task or decision-making, has been shown to vary depending on the time of day (8; 47). This indicates that cognitive processing abilities depend on the time of day (43). We decided to study the effects of time-on-task on mental fatigue, and further explore if the variations in the processing abilities could be linked to variation in mental fatigue due to the time of day.

The first smooth-pursuit based eye-typing system was developed by Lutz et al. (31), using one-point calibration on a 24-inch screen and a 60 Hz eyetracker. Using an ambiguous keyboard design, Lutz et al. (31) achieved a maximum typing speed of 3.41 words per minute (WPM), without training. Zeng & Roetting (51) improved the typing system by introducing word-prediction based on a convolutional neural network, achieving a maximum typing speed of 4.5 WPM. Additional eye-typing assistive systems are detailed in Bafna et al. (2).

**Figure 1.**
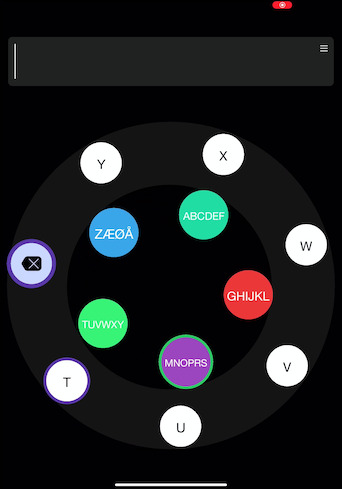
The EyeTell application, with five targets circulating clockwise in the inner circle and seven targets circulating counter-clockwise in the outer circle. The outer circle has the individual letters from T,U,V,W,X and Y, which indicates that the cluster TUVWXY (green target from the inner circle) was selected previously.

EyeTell (2) differs from most of these existing keyboards in two ways – (1) it has been implemented on a tablet, and (2) it does not require any calibration. Other smooth-pursuit typing systems not using individual calibration have been proposed. For instance, Zhe et al. presented a system with a chin-rest setup that would only need one initial calibration from one participant to work for all participants (52)). We conducted an eye-typing task with EyeTell, an application developed for calibration-free eye-typing using smooth-pursuit eye movements on a tablet and without the use of a chin-rest. The eye-typing framework in EyeTell is implemented using an ambiguous Danish keyboard, which allows typing using a small number of on-screen keys (32). The alphabet is divided into clusters of letters and a key on the keyboard denoting a cluster of letters which needs to be selected first, to subsequently select a letter within the cluster. Target letters are circulating counter-clockwise in the outer circle and clusters of letters are circulating clockwise in the inner circle (see Figure 1). To select a target, it should be followed for at least 2 s with a smooth-pursuit eye movement. The process of selection as well as the key performance metrics, such as typing speed and error rate, have been depicted in (2).

In this paper, we report on the effects of mental fatigue, induced by the time of day and cognitive load with time-on-task, on smooth-pursuit eye movements. We designed an experiment requiring cognitive processing during a smooth-pursuit based eye-typing task, to induce mental fatigue. Two levels of task difficulty were generated in a word-recall test, using words of varying difficulty, and thereby generating varying cognitive load. The experiment was repeated in the morning and afternoon, to investigate the effect of time of day on mental fatigue.

Smooth-pursuit has been commonly characterized using the root-mean squared error, or gaze deviation, and ratio of eye velocity and target velocity, termed smooth-pursuit gain. The relationship between attention and eye movements is complex (40; 50; 15), but in general it has been determined that tracking and selecting a single object from multiple stimuli, as in the case of EyeTell, requires focused attention on the target object (6). This increase in attention on the target is accompanied with an increase in smooth-pursuit gain (6). Using a dual-task paradigm, Hutton & Tegally (21) found that divided attention between a non-spatial task and smooth-pursuit task resulted in impairment of the smooth-pursuit and thereby a reduction in smooth-pursuit gain and an increase in the gaze deviation. Similarly, Kosch et al. (26), using the dual task paradigm with an auditory n-back task with numbers and smooth-pursuit eye movement, showed that the gaze deviation was higher while performing the dual task, compared to only a smooth-pursuit task, indicating a reduction in attention. On the other hand, Stubbs et al. (41), used an enhanced target attention experiment, combining the smooth-pursuit task with an n-back task and asking participants if the current moving target color matched the nth most recent moving target color. Enhanced attention to the target, by paying attention to the target and its color, was accompanied with a reduced gaze deviation and an increased velocity gain, but the effect of the velocity gain was not significant.

In this experiment, we have implemented a task of eye-typing using smooth-pursuit interactions on an iPad, without requiring calibration of the iPad screen. The goal of the experiment was to find the mental fatigue effects of time-on-task and time of day on smooth-pursuit eye movement performance characterized by gaze deviation and smooth-pursuit gain. We hypothesized that the smooth-pursuit eye movement performance would reduce as an effect of increased mental fatigue. Moreover, the head movement of the participants was unconstrained, which could cause a variation in the visual angle due to the distance from the iPad; hence we used the circular nature of the target movement and computed angular velocity and angular velocity gain to compute smooth-pursuit gain.

## Methods

In order to find the effect of mental fatigue on smooth-pursuit eye movements, we used EyeTell – a smooth-pursuit based eye-typing application on a tablet in the experiment for the stimuli.

### EyeTell

The application EyeTell consists of targets moving in two concentric circles. At a distance of 40 cm from the screen, a target in the inner circle (diameter: 7 cm) subtended a visual angle of 2^◦^ and moved with a visual velocity of ∼ 4^◦^/s, while a target in the outer circle (diameter: 13.3 cm) subtended a visual angle of 3^◦^ and moved with a visual velocity of ∼ 8^◦^/s. The five targets in the inner circle, separated by 72^◦^ (circular degrees), represent a cluster of letters – ABCDEF, GHIJKL, MNOPRS, TUVWXY and ZÆØA, moving in a clockwise direction. The equi-angular targets in the outer circle, moving in a counter-clockwise direction, depend on the previous selection and contain the individual letters from the last selected target of cluster of letters from the inner circle, as depicted in Figure 1.

A target is selected using smooth-pursuit eye movement following the intended target, and a character is typed by selecting a target from the outer circle. The EyeTell application requires the users to face the screen with both eyes visible, for the application to work, and provides feedback if the eyes are not visible. The distance between the users and the iPad screen was maintained by mounting the iPad to a stand, which was fixed to the table. The application EyeTell and its working performance has been described in further detail in Bafna et al. (2).

### Participants

Thirty-six university students and employees (18 males, 18 females, Age range: 18 – 57 years) volunteered for the experiment. The participants had no previous reports of photosensitive epileptic seizures nor a history of a brain disorder. The Scientific Ethics Committee for the Capital Region in Denmark provided approval of the study protocol (approval number H-18052072), which was conducted in accordance with the Declaration of Helsinki. The participants provided informed consent to take part in the study, and they received a gift card worth 200 DKK.

Six participants were not included in the study due to the following reasons – one participant failed to show up for the second session and the eye-typing data could not be retrieved for five participants. The data analyzed in the experiment was obtained from the remaining 30 participants (15 males, 15 females, Age range: 18 – 57 years).

### Experimental Design

The participants performed the experiment in two sessions – morning and afternoon. The morning session was completed by all participants before 12 pm, and the afternoon session was completed after 12 pm. The participants performed the morning session, on average, at 10 am with 1 hour standard deviation, and they performed the afternoon session, on average at 2:30 pm, with a standard deviation of 1.5 hours. The participants who performed the morning session first completed the afternoon session on the same day. The morning and the afternoon sessions on the same day were separated by a minimum of 3 hours. The participants who performed the afternoon session first completed the morning session on the next day. From the 30 participants whose data was analyzed, 13 performed the afternoon session first, and 17 performed the morning session first.

An experimental session was composed of two trials – with the difficulty levels easy and difficult. The trial with the easy task consisted of memorizing four easy words and eye-typing them; conversely, the trial with the difficult task consisted of memorization of four difficult words and eye-typing. In the first session, after receiving instructions on using the application, the participants practiced eye-typing an easy word for training, and then performed the two trials. During each trial, the participants were given 20 s to read and memorize four target words on a paper presented by the experimenter and were asked to type the words in the same order as they were presented. The target words were then removed from visibility and the participants subsequently typed the words, using the Eye Tell interface.

**Figure 2.**
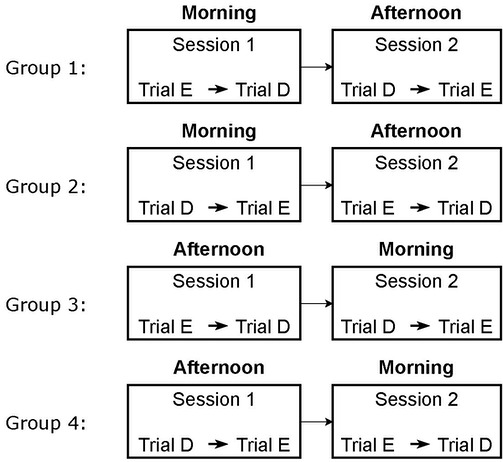
A schematic of the Latin Square experiment design. Trial E indicates an easy task and trial D indicates a difficult task. The order of the easy and difficult was balanced between the sessions, for each participant.

The difficult words were obtained from a list of old and uncommon Danish words obtained from the Danish Language Board (https://dsn.dk/nyt/nyheder/2011/gamle-og-sjaeldne-ord-i-retskrivningsordbogen). Words that were rare and unknown were selected by two native Danish speakers. The easy words were selected as having the same length as the difficult words, from a list of the 360 most common words in Danish (https://lhs2010a.skoleblogs.dk/files/2014/08/De-360-ord-ordliste.pdf). The order of the difficulty levels of the trials was balanced within the two sessions, for each participant, and also within the participants that performed the first session in the morning and afternoon. The participants were randomly assigned to one of the four groups depicted in Figure 2. The experiment was performed in a laboratory blocked from natural light, with stable lighting of ∼ 300 lx, to not influence the participants’ fatigue by daylight.

Before and after each session, the participants reported on their fatigue level. The questions used in the self-reported measures have been derived by combining questions on physical fatigue, mental fatigue, and sleepiness from the Chalder Fatigue Scale and the Visual Analog Scale of Fatigue (VAS-f) and reframed to question about the current fatigue level. The participants also answered questions on the quality of sleep the night before and the number of hours passed since their last caffeine intake. The quality of sleep had four options ranging from ‘very bad’ to ‘very good’. The number of hours since the last caffeine intake had the following options – no caffeine today, more than four hours, between three and four hours, between one and two hours and less than one hour.

The EyeTell application logged data at 30 Hz frequency, which included the typing events of target selection, the path of the targets and the estimated gaze positions. The data was saved locally on the tablet.

The data from the eye-tracker was accessed using a licensed version of Tobii Pro Lab (version 1.152). The eye-tracking data consisted of timestamps, gaze positions and pupil size.

**Figure 3.**
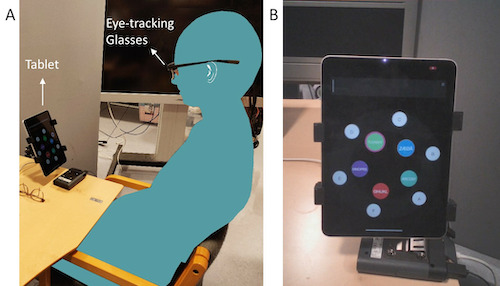
(A) The experimental setup with the eye-tracking glasses and the tablet and (B) a representative snapshot of the camera view from the eye-tracking glasses.

Additionally, we collected eyetracking data using a wearable eye-tracker – Tobii Glasses 2 (sampling frequency: 100 Hz, accuracy: 0.62^◦^± 0.23, precision root-mean square: 0.05^◦^± 0.1; under lighting conditions of 300 lux and a maximum gaze angle of 15^◦^), as depicted in Figure 3A. In the experimental setup, the gaze angle of the outer ring of EyeTell app subtended an angle of 9.4^◦^, assuming that the participant did not move the head considerably. The eye-tracker calibration was performed before starting the experiment.

After the conclusion of the experiment, the participants responded to a web questionnaire on their morningness-eveningness (18).

The independent variables in the experiment were time-on-task, which was based on the trial number, task difficulty level, which could be easy or difficult, session number and time of day, which was morning or afternoon. The dependent variables were measured from the performance on the eye-typing task and the accuracy of the eye movements when compared to the target movement, indicating the performance of the smooth-pursuit eye movement.

### Data Processing

In this section, we have described the data processing steps implemented to standardize the time and location of the target positions, extracted from the EyeTell application, and the gaze positions, recorded using the eye-tracker, and make them comparable to one another.

#### EyeTell target data processing

We manually processed the data from the EyeTell application to synchronize timestamps of the typing events with the data from the eye-tracker. Distinct events, such as letter selection on the EyeTell application, were identified and marked in the eye-tracking data accessible using Tobii Pro Lab. We computed the offset between timestamps of these events between the two data sources, and ensured that the offset was less than 0.5 s. With the targets moving at a constant circular speed of 48^◦^/s, this offset translated to a maximum possible polar angular error of 24^◦^. We expect the synchronization step would not impact the smooth-pursuit gain or gaze deviation, which were averaged over multiple selections.

**Figure 4.**
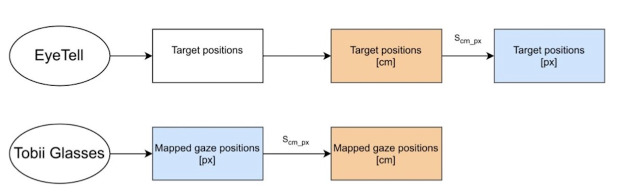
An overview of the EyeTell and eye-tracking data processing with the unit conversions. The blue boxes depict the target and gaze positions in pixels, and which are used for smooth-pursuit onset detection process. The orange boxes depict the target and gaze positions in cm, and which are used to compute the smooth-pursuit performance metrics.

The target positions were extracted from the EyeTell application in the form of x and y coordinates, relative to the screen coordinates, and converted to centimeters. Measurements using a physical ruler and the tablet revealed that 53 screen units equaled 1 cm. The position data of the targets followed a smooth circular path. This conversion is depicted in Figure 4. These data from the EyeTell application, having a frequency of 30 Hz, was linearly extrapolated to match the sampling frequency of the eye-tracker of 100 Hz. The target positions, which were used as the ground truth on the smooth-pursuit eye movement, were extracted and compared to the gaze position, obtained from the eye-tracker.

#### Eye-tracking data processing

The eye tracking data consists of gaze positions, with x and y coordinates, given in pixels, relative to the scenes captured by a camera on the eye-tracking glasses. We captured and saved a representative snapshot of a static scene during every trial. Figure 3B shows an example of a representative snapshot, depicting the camera view of the wearable eye-tracker. Using the assisted mapping feature of Tobii Pro Lab, the gaze positions were mapped onto a 2D coordinate system on this snapshot. This method used image processing techniques and similarity between the static scene from the snapshot and the dynamic video scenes to model the gaze positions on the static snapshot scene. The resulting mapped points were accompanied by a mapping score, between 0 and 1, depicting the similarity between the static scene from the snapshot and the dynamic video scenes throughout the trial. This process was repeated for every trial.

#### Signal processing

The target position data from the EyeTell application, expressed in centimeters, and the gaze position data from the eye-tracker, expressed in pixels, were compared to one-another, to assess the performance of the smooth-pursuit eye movements. To make them comparable, the target position data was converted from centimeters to pixels using a scalar factor – S_cm−px_. The physical measurements of the tablet in the representative snapshot from the camera on the eye-tracking glasses were used to obtain the scaling factor between pixels and centimeters – S_cm−px_, in MATLAB image viewer. The conversion process was carried out for every trial separately and was manually verified by overlaying the target data on the gaze positions. Missing data from the eye-tracker, due to blinks, were linearly interpolated. Further, a hampel filter was applied, replacing gaze positions larger than 2 standard deviations, in a radius of 15 points around the current data point, with a rolling median. The hampel filtered interpolated gaze positions were used further on.

An initial smooth-pursuit classification was performed using velocity and velocity threshold identification (IVVT; 25), with 112^◦^/s as the lower threshold for saccades and 1^◦^/s as the upper threshold to classify fixations. The remaining unclassified data was assigned to the class of smooth-pursuit. The threshold values were empirically realized.

Pearson correlation between the right and left pupil size was computed and was used as a quality criterion. Trials that had a correlation value of 0.75 or less were discarded from the data analysis, due to the low quality of the data.

#### Smooth-pursuit onset detection of the target selection process

Although the EyeTell application selected targets using its estimated gaze positions, the application sometimes selected a target even though the gaze positions did not follow the particular target path, resulting in a false selection. Thus, we used post-hoc analysis of the gaze data and the EyeTell target position data to determine whether the target was selected and if it was, we estimated the onset of the selection.

The gaze position data from the eye-tracker and the target position data from EyeTell, both in pixels, were used to detect the onset of the target selection. The path of the targets and the gaze positions obtained in the time between the previous target selection and the current target selection was analyzed. As the smooth-pursuit eye movements are generated to follow a certain target from multiple targets, it would mean that the gaze positions are closer to the path of the intended target and farther from the path of the adjacent targets. The path of the targets, and thereby the gaze positions, is circular and so computation of polar coordinates from the circular paths enables comparison of the two paths using the polar angles. The polar angle of the gaze positions can match the polar angles from only one target.

The radii and the polar angles for the target positions and the gaze positions were computed. The difference in the polar angles of the gaze positions from that of the target, generating polar angle difference, was used to confirm the target selection and detect the smooth-pursuit onset. If the target was being followed by the gaze, the polar angle difference would be 0^◦^. The polar angle difference was examined with respect to the maximum possible deviation, denoted by the separation between the intended target and the adjacent target. An adjacent target is separated from the intended target by a constant angle; for example, the targets from the inner circle, containing five targets, are separated by 72^◦^. These separation limits between the targets, termed angular limits, were used to denote the limits of the polar angle difference that allowed selection of the intended target. The smooth-pursuit onset was computed as the timestamp when the polar angle difference was within the limit of 0.5 ∗ angular limits for at least 1 s, with the assumption that the smooth-pursuit movement would be accurate directly after the user found and started following the intended target.

The gaze positions between the smooth-pursuit onset and the selection completion constituted as smooth-pursuit eye movements. Finally, as a quality check, only the target selections that contained less than or equal to 30% missing data (due to blinks, etc), where percentage of gaze positions with mapping score below 0.5 was less then 5% and the smooth-pursuit eye movements lasted more than 1 s, were included in the data. The smooth-pursuit eye movements, which had the median polar angle difference greater than (angular limits−20^◦^), were marked as false selections. This threshold was obtained empirically, by observing the false selections which were corrected immediately.

In order to compute the metrics from the smooth-pursuit eye movements, the gaze positions and target positions were converted from pixels to centimeters using the previously used scaling factor - S_cm−px_.

#### Metrics from eye-typing and eye movements

Metrics from three categories were computed and analyzed for effects of mental fatigue – performance, eye movements and self-reported measures. The metrics and their definitions are provided in Table 1. The independent variables used for the analysis were time-on-task, task difficulty level, session number and time of day.

Angular velocity, with the unit ^◦^/s, used in the computation of angular velocity gain, was computed using the polar angles determined previously for both the target and gaze positions, discounting the movements performed during catch-up saccades. The catch-up saccades were determined as the movements classified as saccades by the IVVT algorithm, occurring during the smooth-pursuit movements.

To compute the effect of time-on-task, the eye movement metrics gaze deviation and angular velocity gain were also computed for the extremities on time-on-task. Marginal gaze deviation was computed as the mean gaze deviation of the first 10 selections, if trial 1 was being considered, and as the mean gaze deviation of the last 10 selections, if trial 2 was being considered. Marginal angular velocity gain was computed in a similar manner.

The self-reported measures mental fatigue, sleepiness and physical fatigue were computed on a scale of 1-5. The alertness score was a combination of the sleep quality score and alertness from caffeine effectiveness. The former had a range from -2 to +2, not including 0. The score of +2 indicated good sleep quality and -2 indicated bad sleep quality. The latter had a range from 0, denoting no caffeine in the day, to 4, denoting less than one hour since the last caffeine intake. Alertness score was computed as the sum of these two scores.

The metrics (except alertness score) were further adjusted for the effect of the alertness score using the following formula:

**(1):**
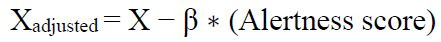


where X_adjusted_ was the adjusted value of the metric, X, and β the regression coefficient of alertness score estimating the relation with the metric, computed over all participants. The adjusted values of the metrics were used to compensate the estimated effects of sleep quality in the night before and caffeine in the body.

**Table 1:** Metrics used to analyze data, and their definitions

Metric (units)	Computation method
	Self-reported measures
Mental fatigue	How difficult is it to concentrate and think clearly?
Sleepiness	How sleepy are you?
Physical fatigue	How would you rate your level of bodily exhaustion?
Alertness score	Sum of the sleep quality of the night before and caffeine effectiveness.
	Performance metrics
Trial duration (min)	Duration of a trial, where a trial starts with the selection of the first character and ends with the selection of the last character
Typing speed (WPM*)	Ratio of the number of words typed in a trial and the trial duration, where one word consisted of five characters, including space
Corrected error rate (%)	Ratio of the backspace selections and the typed characters in a trial
	Eye movement metrics
Gaze deviation (cm)	Root-mean square of the position error between the path created by the target and the gaze positions generated by following the target, for all selections in a trial
Marginal gaze deviation (cm)	Mean gaze deviation of the first 10 selections for trial 1 and last 10 selections for trial 2
Marginal angular velocity gain (no units)	Ratio of the eye movement angular velocity and the target angular velocity of the first 10 selections for trial 1 and last 10 selections for trial 2

Note: *WPM denotes words per minute

### Data Analysis

Linear mixed models (LMM) were used to analyze the data. Random intercepts were employed to model the participant variability. The fixed effects of four independent variables – time of day, time-on-task, task difficulty and session number – were divided into two data types – factors, which were time of day and task difficulty, and numerical variables, which were session number and time-on-task. We performed a variation inflation factor analysis on the independent variables using the statsmodels package (version 0.11.0) in Python 3.6.10 to compute the co-variance between the independent variables. The packages used for the analysis were lmerTest (version 3.1.2; 28) and lme4 (version 1.1.23; 7) in R (version 206 4.0.2; 39).

Multiple comparisons was performed post-hoc using the package multcomp (version 210 1.4.13; 19) and Bonferroni correction was used on the p-values. Effect size computation was based on the method by Nakagawa & Schielzeth (36), using the package r2glmm (version 0.1.2; 22).

#### Self-reported measures

The self-reported measures (adjusted for the alertness score) of mental fatigue, physical fatigue and sleepiness were assessed for the effects of time of day, time-on-task and session number. The alertness score was tested for the effect of time of day, to ensure that the adjustment step for the alertness score did not transfer unintended effects.

#### Performance metrics

The performance metrics – typing speed, trial duration and corrected error rate (adjusted for the alertness score) were tested for effects of task difficulty, time-on-task, session number and time of day.

#### Eye movement metrics

Gaze deviation, computed for the complete trial duration and adjusted for the alertness score, was tested for the effects of task difficulty, time-on-task, session number and time of day. To further test the effect of time-on-task on the eye movement metrics, the data acquired during the first 10 selections was selected for trial 1 and the data acquired during the last 10 selections was selected for trial 2, to compute marginal eye movement metrics. Marginal gaze deviation and angular velocity gain (both adjusted for the alertness score) were tested for the effect of time-on-task using LMM.

## Results

The data analyzed is from 30 participants performing two trials in each of the two sessions, generating four trials per participant. Three of the participants had an inter-pupil correlation lower than 0.75 in all their trials, and their data was removed from the analysis. The subjective data from 108 trials (27*4) was used for analysis. Eye-tracking data from one of the sessions of four participants could not be retrieved and the time synchronization offset for one session for two participants was greater than the threshold 0.5 s, and the performance metrics were available for 96 trials. Finally, the eye movement metrics were computed for the trials where the session contained minimum 10 selections in each trial, not including false selections, with each selection consisting of a maximum of 30% missing data, containing less than 5% gaze positions with a mapping score less than 0.5 and the selection lasting at least 1 s. The eye movement metrics were available for 84 trials from 23 participants. We have reported the data in terms of marginal means and the standard deviations.

We performed variance inflation factor analysis on the independent variables, and found the factors for the variables session, time-on-task, time of day and task difficulty to be 1.5, 1.6, 1.6 and 1.5, respectively, indicating low co-variance between the independent variables.

As a general working of the EyeTell applications, the mean typing speed achieved, including all character selections, was 1.28 WPM ± 0.33. The application made false selections for 19.5% ± 12.8 of the targets selected by the user.

Twenty-three participants responded to the morningness-eveningness questionnaire, and were classified most-commonly as intermediate (16), followed by evening-type (5) and morning-type (2).

### Self-reported measures

We hypothesized that the self-reported measures mental and physical fatigue and sleepiness (after adjusted from alertness score) would be higher in the afternoon, when compared to the morning, and higher after the experiment relative to before the experiment.

The regression coefficient of alertness score, β, was found to be -0.09, -0.1 and -0.1 for mental fatigue, physical fatigue and sleepiness, respectively. The negative coefficients exhibit the inverse relation of the self-reported measures with the alertness score.

**Figure 5.**
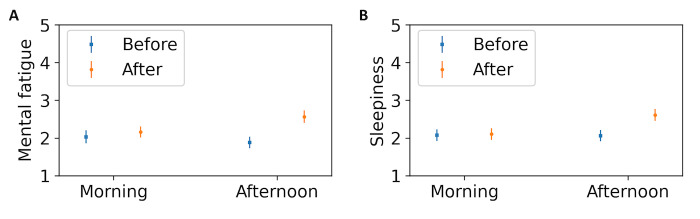
Self-reported measures recorded before and after the experiment, as a function of the time of day – (A) Mental fatigue and (B) Sleepiness. Error bars indicate standard deviation.

The marginal means of mental fatigue showed no effect of the time of day (morning: 2.09 ± 0.11, afternoon: 2.22 ± 0.13), as seen in Figure 5A. The mental fatigue increased after the experiment, compared to before (before: 1.98 ± 0.12, after: 2.35 ± 0.12). LMM revealed the difference before and after the experiment to be significant (χ^2^(1) = 5.75, p < 0.05, η^2^ = 0.04). The effects of time-on-task were revealed to have significance on multiple comparisons (p < 0.05).

Physical fatigue showed effect of neither the time of day (morning: 2.23 ± 0.12, afternoon: 2.13 ± 0.10) nor the time-on-task (before: 2.14 ± 0.10, after: 2.25 ± 0.11).

The marginal means for sleepiness were higher in the afternoon compared to the morning (morning: 2.08 ± 0.11, afternoon: 2.34 ± 0.12), however the effect was not significant (p>0.1). The sleepiness was higher after the experiment, compared to before (before: 2.09 ± 0.11, after: 2.35 ± 0.11), as seen in Figure 5B. The effect was tested using LMM and was found to be significant (χ^2^(1) = 5.01, p < 0.05, η^2^ = 0.06). There was also an interaction between the time-on-task and session number, depicting a higher increase in sleepiness after the experiment, in session 1 compared to session 2 (χ^2^(1) = 5.76, p < 0.05,η^2^ = 0.04). The effects of time-on-task and its interaction with session number were revealed to have significance on multiple comparisons (p < 0.05).

The marginal means for the alertness score were slightly higher in the afternoon compared to the morning (morning: 2.15 ± 0.25, afternoon: 2.48 ± 0.19). The effect was tested using LMM and found to not be significant (p > 0.1).

**Table 2:** Linear mixed effects model results of the metrics adjusted for alertness indicating the main effects of time-on-task, session and task difficulty

Feature	Time-on-task			Session			Task difficulty		
	β ± S.E.	χ^2^(1)	η^2^	β ± S.E.	χ^2^(1)	η2	β ± S.E.	χ^2^(1)	𝜂^2^
	Performance metrics								
Trial duration	-70.4 ± 24.5	7.78^a^	0.04	-147.6 ± 25.3	28.5^c^	0.15	-	-	-
Typing speed	0.14 ± 0.05	7.87^b^	0.05	0.14 ± 0.05	8.22^b^	0.06	0.21 ± 0.05	16.3^c^	0.11
Corrected error rate	-4.68 ± 1.50	9.12^b^	0.08	-3.43 ± 1.52	4.94^d^	0.04	-	-	-
	Eye movement metrics								
Gaze deviation	-	-	-	-	-	-	0.17 ± 0.08	4.76	0.03
Marginal gaze deviation	0.47 ± 0.12	13.8^c^	0.14	-	-	-	-	-	-
Marginal angular velocity	-0.08 ± 0.03	7.14^a^	0.08	-	-	-	-	-	-

^a^ p < 0.05 ^b^ p < 0.01 ^c^ p < 0.001 ^d^ Bonferroni correction resulted in the feature to be non-significant (p > 0.05)

Trial duration was higher for the easy task (easy: 8.52 min ± 0.42, difficult: 7.69 min ± 0.38) but the effect was not significant (p > 0.1). Trial duration decreased in the second trial by 1.17 min ± 0.39. The duration of the trial reduced in the second session by an average of 2.58 min ± 0.59. The effects of time-on-task and session number were revealed to have significance when analyzed using LMM (p < 0.05). The LMM model parameters are provided in Table 2. The marginal mean duration of session 1 was 19.89 min ± 1.70 min, and for session 2 it was 14.57 min ± 0.98 min.

The typing speed of the eye-typing tasks increased by 0.13 WPM ± 0.04 in the second trial and by 0.15 WPM ± 0.06 in the second session, compared to the first. The typing speed was most affected by the task difficulty and was higher for easy tasks compared to difficult (easy: 1.36 WPM ± 0.05, difficult: 1.15 WPM ± 0.04). The effects were found to be significant on LMM analysis. The LMM model parameters are provided in Table 2.

The last performance metric – corrected error rate – had no effect of the task difficulty (easy: 16.4% ± 1.4, difficult: 14.0% ± 0.9). However, it was found to reduce with time-on-task by 4.68% ± 1.13 and lowered by 3.19% ± 1.75 during the second session compared to the first. Using LMM, the effects of time-on-task were found to be significant, on multiple comparisons, c.f. Table 2.

### Eye movement metrics

We hypothesized that gaze deviation would show an effect of the task difficulty. Additionally, we computed marginal eye movement metrics, and compared the metrics from the first 10 selections of trial 1 to the last 10 selections of trial 2 and hypothesized that the marginal gaze deviation and the marginal angular velocity gain would increase and decrease, respectively, with time-on-task.

The metrics were adjusted for effects of the alertness score and the coefficient, β, obtained were -0.05, -0.02 and 0.006 for gaze deviation, marginal gaze deviation and marginal angular velocity gain, respectively. The low coefficients indicate that the alertness score did not have a big impact on these metrics.

Gaze deviation was found to be lower for the difficult tasks (easy: 2.82 cm ± 0.08, difficult: 2.65 cm ± 0.06). The effect was found to be significant using LMM, the parameters of which are provided in Table 2. The effects of the task difficulty were found to be not significant on multiple comparisons (p > 0.05).

**Figure 6.**
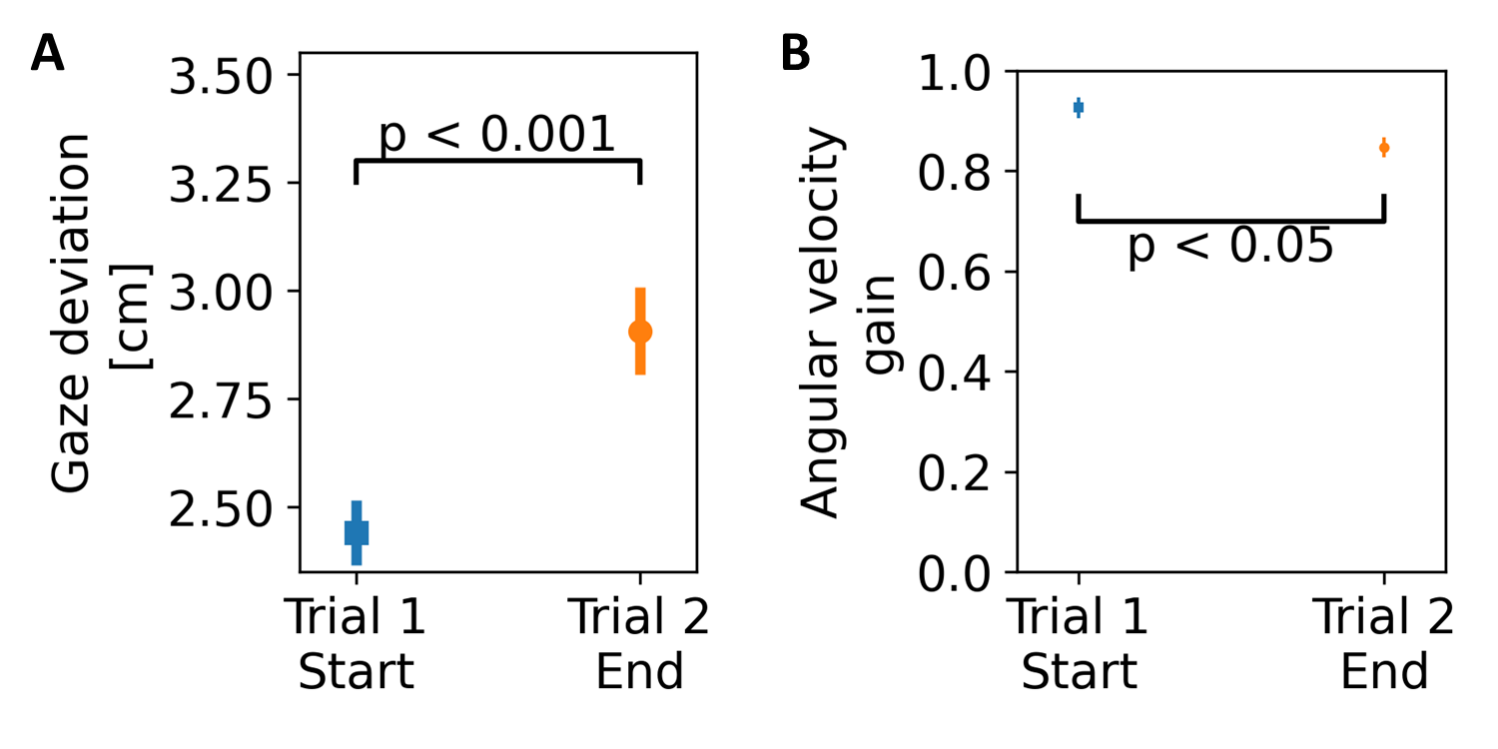
Eye movement metrics as a function of the time-on-task – (A) marginal gaze deviation and (B) marginal angular velocity gain. Error bars indicate standard deviation.

Marginal gaze deviation increased as a function of time-on-task by 0.47 cm ±0.09 (see Figure 6). The effect was found to be significant using LMM (see Table 2) and indicated a reduction in the ability to smoothly follow the target. The effects of time-on-task were revealed to have significance on multiple comparisons (p < 0.05).

Marginal angular velocity gain reduced at the end of trial 2, compared to the beginning of trial 1, by 0.08 ± 0.02. The effect of time-on-task was found to be significant using LMM (see Table 2) This indicated that compared to the target velocity, the velocity of the eye movements slowed down as a function of time-on-task. The effects of time-on-task were revealed to have significance on multiple comparisons (p < 0.01).

## Discussion

In the present study, we investigated the effect of time-on-task and time of day on mental fatigue, and if the variation in mental fatigue could be observed in smooth-pursuit eye movements. We performed an experimental procedure using working memory combined with eye-typing using smooth-pursuit eye movements, to induce mental fatigue by time-on-task and varying cognitive load and repeated the experiment in the morning and the afternoon, to observe the effect of time of day on mental fatigue. The self-reported mental fatigue increased after the experiment compared to before, however, the effect of the time of day was not significant. The smooth-pursuit eye movements performed for eye-typing exhibited an increase in the mental fatigue level; the deviation from the target path increased with time-on-task, while the angular velocity gain decreased indicating slowing of the gaze velocity when compared to the target velocity.

As observed from the data, the time of day did not have an effect on the mental fatigue level. The responses on the morningness-eveningness questionnaire classified most of the responders as intermediate-type, and thereby might have no clear effect on the mental fatigue level at the different times of day. Figure 5(a) indicates that mental fatigue recorded before the experiment reduced in the afternoon, compared to the morning. The alertness score was slightly higher in the afternoon compared to the morning, although it was not significantly so, suggesting that the participants perhaps may have prepared to counteract fatiguing effects by ingesting caffeine before the experiment in the afternoon. Moreover, a majority of the participants were from the age group 18-37, who tend to be evening- and neutral-types, in terms of morning-evening type of personality (49). However, the increase in mental fatigue due to the task was higher in the afternoon (as observed in Figure 5(a)). This indicates that in the afternoon, although the participants were not tired before the experiment, performing the tasks could have increased the mental fatigue level. Similar to muscle fatigability, which is the failure to maintain force during sustained contraction, mental fatigability is the failure to maintain attention during sustained cognitive processing and could explain the phenomenon observed in this study. The effect, however, was found to be not significant using linear mixed models, which could be due to the experiment consisting of only two trials, while an increased number of trials might have increased the effect of mental fatigue due to time-on-task in the afternoon.

Although the number of trials was only two and the experiment duration was short, the experiment resulted in an increase in the mental fatigue level. The novelty of the smooth-pursuit task and the memory task with four words could have caused this increase in mental fatigue. Kahneman & Beatty (23) showed that the memory load generated by verbal recall of four frequently used words was high and could be detected in the pupil dilation. The combination of the memory load for the duration of eye-typing with the novel eye-typing methods increased the mental fatigue level on the participants.

We detected an impaired smooth-pursuit performance in response to increase in mental fatigue, during an eye-typing task. Reduced attention was assumed to cause the impairment, which has been observed in numerous studies (41; 21; 6). Gaze deviation, in our study, was also studied with respect to the task difficulty and was found to decrease during the difficult task. This decrease in gaze deviation during a more difficult task indicated increased focused attention to the task, and consequently the target, consistent with the results on a dual-task study by Stubbs et al. (41), where enhanced attention reduced the variability between the gaze and target positions.

This experiment was performed in a head-free design by the participants. Head movements, specifically the vestibulo-ocular reflex can have an effect on the smooth-pursuit eye movements (3). Moreover, when the users move their heads and eyes simultaneously to achieve the pursuit movement, the gaze positions might not convey the complete movement data and may not match the target positions (46). The smooth-pursuit parameters used to detect mental fatigue were the correctly selected targets, which might have mitigated some effects of the head movements in the data analysis.

The time synchronization step between the data from EyeTell and the eyetracker led to a maximum uncertainty in the gaze polar angles of 24^◦^. We have no reason to believe that the gaze polar angles were always lagging or always leading, compared with the target polar angles. Moreover, the metrics computed during the smooth-pursuit eye movements were averaged over several selections, and the offset could not influence the metrics in one direction or bias the results. Moreover, the effect of using the Tobii Glasses with an accuracy of 0.62^◦^ would be minimal on the smooth-pursuit metrics, due to the amount of difference observed in the marginal smooth-pursuit metrics.

We decided to control for the effect of caffeine using data analysis by correcting the metrics for the alertness score, which accounted for caffeine intake and the sleep quality from the night before the experiment. Most of the studies on mental fatigue measurement restrict substance use before the experiment, to not confound the data (17; 33). However, it can be argued that this restriction might have an adverse effect on habitual caffeine drinkers, causing withdrawal symptoms even from short duration of restriction (38). The most common method to control for a confounding factor is to add the factor in the regression model (34). However, we have used mixed models in our analysis, which are reduced so that all the factors in the final model have a significant effect on the metric modeled. The alertness score did not have a significant effect on the metrics, and to compensate for it, we decided to adjust the metrics to remove the effect of the alertness score.

We should note that we recorded an increase in both the mental fatigue and sleepiness measures due to time-on-task. Although the concepts are distinct, mental fatigue and sleepiness share a relationship with arousal and no objective physiological data has been found yet, which can distinguish between them (20). Although the eye-typing task was self-paced and the lighting in the laboratory was high, it is possible that strain on the eyes due to an eye-interactive task was inferred as sleepiness by the participants, who are not habituated to using voluntary eye movements to type on an iPad, instead of their hands. Further research is required to determine physiological signals that can distinguish mental fatigue and sleepiness.

Further research is also required to examine the smooth-pursuit performance metrics and the effects of increased time spent on the task with different levels of cognitive load, and the consistency and reliability of the metrics to detect mental fatigue under varying conditions. Research is furthermore required to find the validity of the metrics for smooth-pursuit-based tasks other than eye-typing.

There are some limitations to the study performed. The application EyeTell was a first of its kind, and at a nascent stage. At 19% false selections, it selected a target not followed by the user often, which can be attributed to the lack of calibration before use. The false selections could be improved by using absolute calibration or by reducing the number of targets visible on the screen. Another limitation is that most of the participants were less than 40 years old, which could account for the absence of fatigue effect observed due to time of day. A limitation of the study is that the application EyeTell is targeted for people with motor disabilities, but the study was conducted with the able-bodied population. The experiment needs to be repeated with people with motor disability, to establish that the same metrics can be used to detect mental fatigue.

The implications of the feasibility of detecting mental fatigue using an iPad are enormous. The additional data collected using the wearable eye-tracker in our experiment could be implemented using the iPad, the latest version of which has an infra-red camera. This additional data would provide further insights into the eye movements. This experiment has depicted the feasibility of detecting mental fatigue using smooth-pursuit eye movements, performed for eye-typing interactions. The technique of smooth-pursuit could be implemented in other screens with cameras and measurement of mental fatigue indications could reach workers in offices, students in classrooms and anyone with a screen available. Using the smooth-pursuit technique on a personal device such as a personal tablet could use calibrated gaze positions, which would improve the performance on an application such as EyeTell. For practical uses of the technique of mental fatigue measurement in offices or schools, research would be required to investigate smooth-pursuit based tasks other than eye-typing tasks. Since the smooth-pursuit performance metrics of gaze deviation and angular velocity gain are independent of the task of eye-typing, different games could be developed, which could be more engaging and easily implemented in daily lives.

While there are multiple commercial assistive systems which try to incorporate multiple services in the same customized device, applications like EyeTell could be a starting point to incorporate different services in a tablet, a device available to everyone, with and without a motor disability. To utilize the smooth-pursuit metrics for detection of mental fatigue for people with motor disability, further research is required. Where would these applications be most useful during practical use and how would the additional information on mental fatigue be exploited?

In conclusion, the current study has illustrated a novel method depicting the feasibility of detecting mental fatigue, during eye-typing, using smooth-pursuit eye movements in conjunction with eye-typing. The effect of time-on-task on mental fatigue was observed using impairments in the smooth-pursuit performance, denoted by the increase in gaze deviation and decrease in angular velocity gain. These results indicate that smooth-pursuit eye movements, performed for eye-typing, are sensitive to mental fatigue, and it is feasible to measure the mental fatigue.

### Ethics and Conflict of Interest

The author(s) declare(s) that the contents of the article are in agreement with the ethics described in http://biblio.unibe.ch/portale/elibrary/BOP/jemr/ethics.html and that there is no conflict of interest regarding the publication of this paper.

### Acknowledgements

The research has partly been supported by the EU Horizont 2020 ReHyb project and The Bevica Foundation. Thanks to Elias Pedersen and Frederik Neble for advice and software support.
